# Insights from an early career faculty on peer review

**DOI:** 10.1063/4.0001103

**Published:** 2025-10-27

**Authors:** Chrystal Starbird

**Affiliations:** University of North Carolina at Chapel Hill, Chapel Hill, NC, USA

## Abstract

This session will share insights from an early career faculty into their experiences with peer review in both industry and academia, and to how their participation in peer review has grown as their career progressed. The speaker will share an overview of their experience in peer review as a trainee and how this experience has changed as they transitioned into their faculty career, going from joint peer review to joining editorial advisory boards . Topics covered will include how to find opportunities, ways to expand your network for peer review, how to establish yourself as a reliable peer reviewer, and how to leverage your network to pitch ideas to editors.

